# Hilar cholangiocarcinoma associated with pancreaticobiliary maljunction, pancreas divisum, and a type V right hepatic duct variant: a case report

**DOI:** 10.3389/fsurg.2026.1931578

**Published:** 2026-10-19

**Authors:** Ruipeng Dong, Peipei Cong, Guangjun Shi

**Affiliations:** 1 Department of Hepatobiliary Surgery, Qingdao Municipal Hospital, Qingdao, China; 2 Dalian Medical University, Dalian, China

**Keywords:** bile duct variation, case report, hilar cholangiocarcinoma, pancreas divisum, pancreaticobiliary maljunction

## Abstract

This case describes hilar cholangiocarcinoma with pancreaticobiliary maljunction (PBM), pancreas divisum, and a type V right hepatic duct variant. Preoperative computed tomography (CT) with three-dimensional (3D) reconstruction and magnetic resonance cholangiopancreatography (MRCP) clarified the complex ductal anatomy and informed reconstruction. After local extrahepatic bile duct resection with hilar lymphadenectomy, the patient initially recovered uneventfully. At 5 months, CA19-9 was 4,360 U/mL, CEA was within the reference range, and imaging raised concern for recurrence. The patient died at home 6 months after surgery; multiorgan failure was reported, but no autopsy was performed. This case emphasises the need for detailed preoperative mapping of coexisting biliary and pancreatic ductal variants to guide operative planning and biliary reconstruction. It also illustrates the potential oncological limitations of local extrahepatic bile duct resection in the setting of incomplete disease staging.

## Introduction

Hilar cholangiocarcinoma is a challenging malignant biliary tumour with a generally poor prognosis. Surgery remains the only potentially curative treatment, but the operative strategy is highly dependent on local biliary anatomy. Pancreaticobiliary maljunction (PBM) is a congenital anomaly in which the pancreatic and bile ducts join outside the duodenal wall, allowing reciprocal reflux of pancreatic juice and bile. This abnormal reflux is associated with chronic mucosal injury and an increased risk of biliary tract malignancy (1, 2).

Pancreas divisum is the most common congenital variation of the pancreas and reflects failure of fusion between the ventral and dorsal pancreatic ducts. Previous reports suggest that pancreas divisum may coexist with bile duct confluence variants, although the association is not fully established (3). Variations at the hepatic hilum are also clinically important because they influence the extent of liver resection and the method of biliary reconstruction (4).

We report a patient with hilar cholangiocarcinoma complicated by PBM, pancreas divisum, and a type V right hepatic duct variant (4) (Figure 1). The report focuses on the contribution of preoperative anatomical mapping to drainage and reconstruction, the limitations of applying the conventional Bismuth-Corlette classification to anomalous hilar anatomy, and the oncological limitations of local bile duct resection.

**Figure 1:**
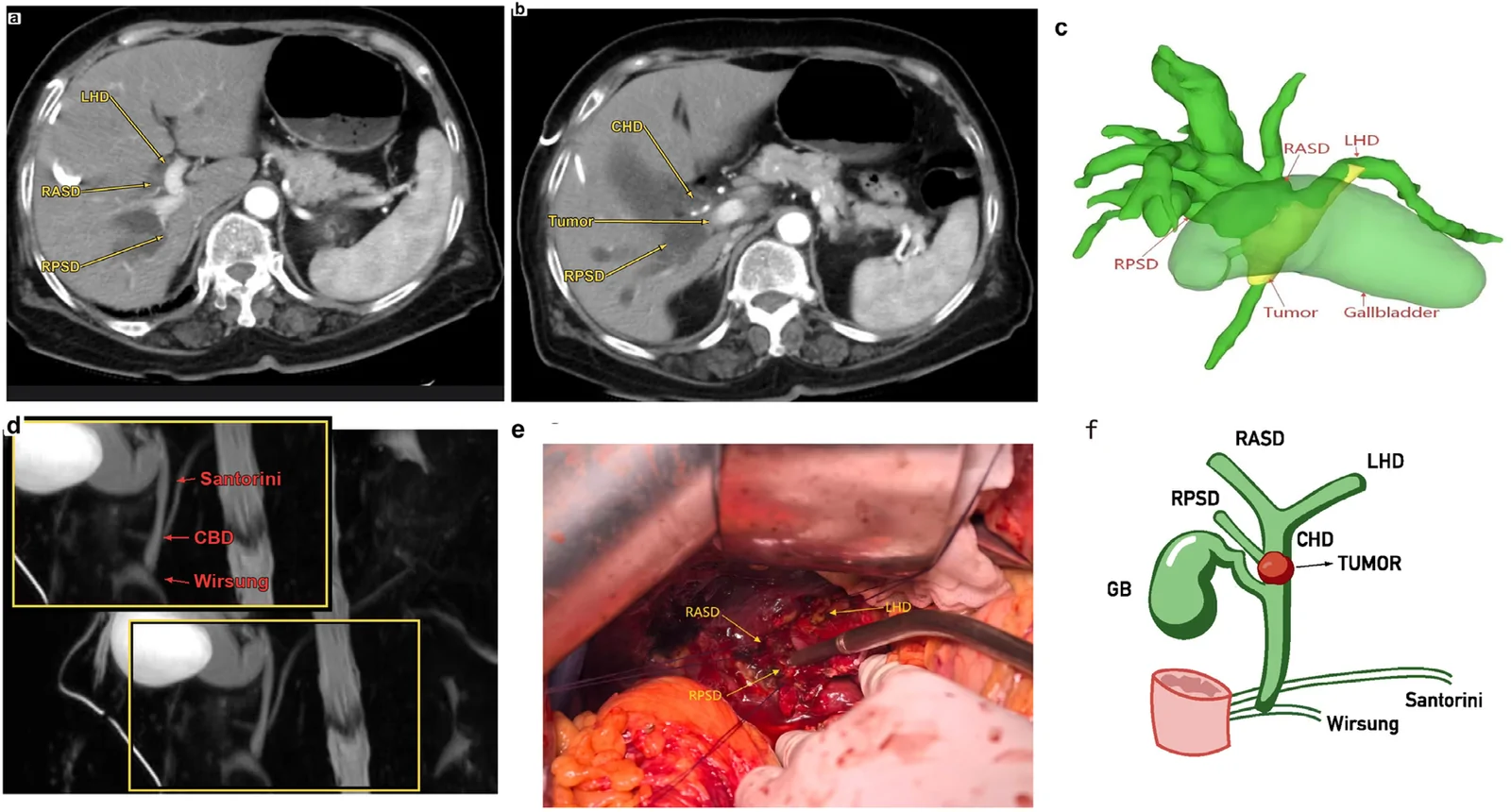
Cross-sectional, reconstructed, intraoperative, and schematic representations of the hilar tumour and pancreaticobiliary anatomy. **(a)** Axial contrast-enhanced CT showing the LHD, RASD, and RPSD. **(b)** Axial contrast-enhanced CT showing the hilar tumour, CHD, and RPSD. **(c)** 3D reconstruction demonstrating the hilar tumour and type V right hepatic duct variant. **(d)** MRCP showing the pancreaticobiliary anatomy; the inset at the upper left is a 1.1-fold magnified view reframed to centre the annotated pancreaticobiliary duct region. **(e)** Intraoperative view showing the preserved ductal stumps before biliary reconstruction. **(f)** Patient-specific schematic of the hilar biliary and pancreatic ductal anatomy, showing the RASD, RPSD, LHD, CHD, gallbladder, hilar tumour, and separate dorsal (Santorini) and ventral (Wirsung) pancreatic ducts, consistent with pancreas divisum. The schematic is not to scale. CHD, common hepatic duct; CBD, common bile duct; GB, gallbladder; LHD, left hepatic duct; MRCP, magnetic resonance cholangiopancreatography; RASD, right anterior sectoral duct; RPSD, right posterior sectoral duct.

## Case presentation

### Patient information

A 71-year-old patient was admitted with progressive jaundice for 15 days on 21 February 2025. The patient also reported intermittent upper abdominal pain. There was no fever, nausea, vomiting, or significant weight loss. The available medical and family histories were unremarkable. No additional socioeconomic or psychosocial information was documented in the available record.

### Clinical findings

Physical examination showed jaundice of the skin and sclera, a soft abdomen, upper abdominal tenderness without rebound tenderness, and a negative Murphy's sign.

### Diagnostic assessment

Ultrasound-guided percutaneous transhepatic biliary drainage (PTCD) of the right anterior sectoral duct (RASD) was performed on 21 February. Laboratory tests revealed an albumin level of 31.26 g/L, total/direct bilirubin 237.60/113.90 μmol/L, alanine/aspartate aminotransferase 174.33/140.09 U/L, and CA19-9 365.4 U/mL, consistent with obstructive jaundice and suggesting malignancy. Serum amylase was normal. Persistent dilatation prompted additional PTCD of the right posterior sectoral duct (RPSD) on 5 March. By 8 March, these values were 32.57 g/L, 73.90/34.90 μmol/L, and 63.94/66.28 U/L, respectively.

Contrast-enhanced CT with 3D reconstruction showed a nodular mass at the hepatic hilum with proximal bile duct dilatation. The RASD joined the left hepatic duct to form the common hepatic duct, whereas the RPSD entered the common hepatic duct extrahepatically, consistent with a type V variant. The tumour was centred at the RPSD-common hepatic duct junction and involved the RPSD. MRCP demonstrated a complex pancreaticobiliary confluence anomaly and pancreas divisum. Intraoperative bile analysis showed markedly elevated amylase in gallbladder bile (124,576 U/L) and bile duct bile (7,776 U/L), supporting pancreaticobiliary reflux.

MRCP showed a 13.36-mm common channel and a pancreaticobiliary junction outside the duodenal wall, meeting the imaging definition of PBM. Markedly elevated amylase concentrations in gallbladder and bile duct bile provided additional support.

The final diagnosis was hilar cholangiocarcinoma with PBM, pancreas divisum, and a type V right hepatic duct variant. The anomalous hilar confluence prevented reliable assignment of a Bismuth-Corlette type; the tumour could not be classified confidently as type I or type IIIa. We therefore describe the involved ducts directly. Chest CT showed no metastasis. PET-CT and lower abdominal and pelvic CT were not performed, so distant metastatic disease was not fully assessed.

### Therapeutic intervention

Before treatment, the patient personally signed a written authorisation designating named family members to receive medical information, make treatment decisions, and sign procedure-specific consent documents on the patient's behalf. The procedure-specific consent form for open biliary surgery documented the proposed operation, potential risks and complications, alternative treatment options, and the treating physicians' disclosure; it was signed by the patient's son, who was one of the named authorised representatives. The available record does not contain a contemporaneous note explicitly comparing local extrahepatic bile duct resection with hepatectomy and possible pancreaticoduodenectomy or recording a refusal of the more extensive procedures. It also does not contain a structured assessment of the patient's decision-making capacity or explicit documentation of the patient's own understanding of, or preference regarding, the extent of surgery.

The anomalous hilar duct anatomy prevented reliable application of the Bismuth-Corlette classification. The patient also had impaired liver function and poor nutritional status, which were considered to increase the risk of hepatectomy. Local extrahepatic bile duct resection with hilar lymphadenectomy was selected in view of these factors. A multidisciplinary tumour-board discussion was not documented. The available chart also does not establish whether more extensive resection was discussed with the patient or family or declined.

Because the RPSD entered the extrahepatic common hepatic duct separately, it required an independent transection and a separate hepaticojejunostomy; the remaining hilar duct was reconstructed through a second anastomosis.

On 11 March 2025, the patient underwent *en bloc* local extrahepatic bile duct resection with hilar lymphadenectomy. Separate hepaticojejunostomies were created for the RPSD stump and the remaining hilar duct. No hepatic, caudate lobe, or vascular resection was performed. The proximal ducts were transected at the hepatic hilum, and the distal duct was transected within the intrapancreatic segment of the common bile duct. Frozen sections of both preserved proximal duct stumps and the distal margin were negative. No peritoneal or omental nodules or tumour rupture were observed. Bile spillage was contained with gauze and managed by warm saline irrigation.

### Pathological findings

Histopathology showed a 1.2 × 1.0 × 0.6 cm moderately differentiated extrahepatic bile duct adenocarcinoma invading the adventitia to a depth of 6 mm, with perineural invasion and tumour emboli. The final proximal and distal ductal margins were negative, and two group 8 lymph nodes were negative. The reported pathological stage was pT2aN0. The clinical metastatic status was recorded as cM0 based on the available imaging; however, PET-CT and lower abdominal and pelvic CT were not performed, and distant metastatic disease was therefore not fully assessed. Immunohistochemistry showed CK7(+), CK8(+), CK19(+), EMA(+), EGFR(+), MUC5AC(+), MUC6(+), and beta-catenin(+); Ki-67 labelling was 20%, and p53 showed a wild-type pattern. The gallbladder showed chronic cholecystitis only.

### Follow-up and outcomes

Recovery was uneventful, and the patient was discharged on 27 March 2025 (postoperative day 16). Adjuvant therapy was declined by the designated family representatives; the available record does not separately document the patient's personal preference. At 5 months after surgery, CA19-9 was 4,360 U/mL, and CEA was within the laboratory reference range. CT showed an abnormally enhancing hilar lesion and multiple nodules in the abdominal cavity, intra-abdominal fat planes, and greater omentum, considered radiologically suspicious for recurrence. Some abdominal and retroperitoneal nodes were also suspicious. These findings were not pathologically confirmed. The patient died at home 6 months after surgery. Multiorgan failure was reported; no autopsy was performed.

## Clinical timeline

Table 1.

**Table 1:** Clinical timeline of the patient with hilar cholangiocarcinoma.

Date	Clinical event
6 February 2025	Approximate onset of progressive jaundice.
21 February 2025	Admission; PTCD of the right anterior sectoral duct.
22 February 2025	Albumin 31.26 g/L; total/direct bilirubin 237.60/113.90 μmol/L; ALT/AST 174.33/140.09 U/L.
5 March 2025	Additional PTCD of the right posterior sectoral duct.
8 March 2025	Albumin 32.57 g/L; total/direct bilirubin 73.90/34.90 μmol/L; ALT/AST 63.94/66.28 U/L.
11 March 2025	Local extrahepatic bile duct resection with hilar lymphadenectomy, negative proximal and distal ductal margins, and separate hepaticojejunostomies.
27 March 2025	Discharged on postoperative day 16.
5 months postoperatively	CA19-9 was 4,360 U/mL, and CEA was within the laboratory reference range. CT showed radiologically suspected hilar and intra-abdominal recurrence, including omental nodules and suspicious nodes.
6 months postoperatively	The patient died at home; multiorgan failure was reported, and no autopsy was performed.

## Discussion

This case is notable for the coexistence of three clinically relevant anatomical problems: PBM, pancreas divisum, and a hilar bile duct confluence variant. PBM is a recognised high-risk condition for biliary tract cancer because the abnormal junction permits reflux of pancreatic juice into the biliary tree, causing chronic epithelial injury, repeated repair, and malignant transformation (1, 2). The elevated bile amylase levels in this case were consistent with significant pancreaticobiliary reflux.

Pancreas divisum may further complicate the anatomical picture by altering the expected relationship between the dorsal and ventral pancreatic ducts and the common channel. In the present patient, the coexistence of PBM and pancreas divisum increased the need to map the distal pancreaticobiliary junction before transection and reconstruction. Although the precise developmental relationship between pancreas divisum and hilar bile duct variants remains uncertain, congenital anomalies arising during the same embryologic period may coexist (3). This case also reinforces the value of MRCP and contrast-enhanced CT with 3D reconstruction for preoperative planning. When PBM or other ductal anomalies are suspected, imaging should extend beyond tumour staging to include a detailed map of the distal pancreaticobiliary junction and the hilar confluence (5). Relying on the Bismuth-Corlette classification alone is insufficient when additional congenital anomalies are present.

The bile duct confluence at the hepatic hilum has major operative consequences. Chaib et al. classified right hepatic duct variants into five types, and a type V configuration, in which a right sectoral duct drains separately into the extrahepatic biliary tree, changes the transection plane and reconstruction plan (4). For surgeons, the key issue is the actual three-dimensional biliary anatomy and its implications for resection and reconstruction. In most resectable hilar cholangiocarcinomas, hepatic and caudate resection depends on tumour extent, vascular involvement, margin feasibility, and future liver remnant (6). In this patient, the anomalous confluence precluded reliable Bismuth-Corlette classification, while impaired liver function and poor nutritional status were considered to increase hepatectomy risk. The operation should therefore be described as local extrahepatic bile duct resection with hilar lymphadenectomy, not as standard radical resection.

The consent documents establish that the patient had provided written authorisation for named family representatives and that the patient's authorised son signed the procedure-specific consent. However, the retrospective record contains neither a structured assessment of the patient's decision-making capacity nor explicit documentation of the patient's own understanding of, or personal preference regarding, local versus more extensive resection. It also contains no contemporaneous note explicitly comparing these operative strategies or recording a refusal of the more extensive procedures. The extent of the patient's direct participation in this treatment decision therefore cannot be determined retrospectively.

The patient died at home 6 months after surgery, and multiorgan failure was reported. At 5 months, imaging had raised concern for recurrence, but no lesions were pathologically confirmed, and no autopsy was performed. Neither the cause of death nor a causal link between suspected recurrence and death can therefore be established. Potential explanations for early radiological recurrence include occult disease missed by incomplete staging, aggressive tumour biology reflected by adventitial and perineural invasion, the oncological limitations of local resection, or intraoperative dissemination. A relationship to the PTCD tract cannot be established. Interpretation is also limited by examination of only two lymph nodes, incomplete distant staging, and presentation-derived CT panels without coronal or sagittal reformations. These constraints preclude conclusions about the cause of recurrence or comparative oncological effectiveness.

## Conclusion

In patients with suspected PBM or pancreas divisum, careful preoperative assessment of both the distal pancreaticobiliary junction and the hilar bile duct anatomy is essential. Multimodality imaging can reveal surgically important variants that directly influence the extent of resection and the reconstruction strategy in hilar cholangiocarcinoma. Anatomical precision, however, cannot replace complete staging or standard oncological resection principles.

## Patient perspective

A direct patient perspective could not be obtained after the patient's death. No contemporaneous written statement describing the patient's experience or treatment preferences was available. Socioeconomic and psychosocial details were not documented in the available record.

## Data Availability

The original contributions presented in the study are included in the article/Supplementary Material, further inquiries can be directed to the corresponding author.
